# Real-time PCR of the mammalian hydroxymethylbilane synthase (HMBS) gene for analysis of flea (*Ctenocephalides felis*) feeding patterns on dogs

**DOI:** 10.1186/1756-3305-5-4

**Published:** 2012-01-04

**Authors:** Chengming Wang, Jane Mount, Jamie Butler, Dongya Gao, Euisun Jung, Byron L Blagburn, Bernhard Kaltenboeck

**Affiliations:** 1 Department of Pathobiology, College of Veterinary Medicine, Auburn University, Auburn, AL 36849-5519, USA; 2 Ross University School of Veterinary Medicine, PO Box 334, Basseterre, St. Kitts, West Indies

**Keywords:** Flea, Dog, *Ctenocephalides felis*, feeding, PCR

## Abstract

**Background:**

Precise data on quantitative kinetics of blood feeding of fleas, particularly immediately after contact with the host, are essential for understanding dynamics of flea-borne disease transmission and for evaluating flea control strategies. Standard methods used are inadequate for studies that simulate early events after real-life flea access to the host.

**Methods:**

Here, we developed a novel quantitative polymerase chain reaction targeting mammalian DNA within fleas to quantify blood consumption with high sensitivity and specificity. We used primers and fluorescent probes that amplify the hydroxymethylbilane synthase (HMBS) gene, an evolutionary divergent gene that is unlikely to be detected in insects by mammalian-specific primers and probes. To validate this assay, fleas were placed on dogs, allowed to distribute in the hair, and removed at specific time points with single-use combs. Fleas were then immediately homogenized by vigorous shaking with ceramic beads in guanidinium-based DNA preservation buffer for DNA extraction.

**Results:**

The specificity of this assay was ascertained by amplification of canine, feline and equine blood with differential product melting temperatures (*T*_m_), and lack of amplification of bovine and porcine blood and of adult fleas reared from larvae fed with bovine blood. Sensitivity of the assay was established by limiting dilution and detection of single copies of HMBS DNA equivalent to 0.043 nL blood. Application of the assay indicated that after 15 minutes on a dog, male and female fleas had ingested low, but similar amounts of approximately 1.1. nL blood. Saturation uptake of 118 and 100 nL blood per flea was found at 30 and 60 min on the dog, respectively.

**Conclusions:**

The HMBS PCR method developed here offers the advantages of both exquisite sensitivity and specificity that make it superior to other approaches for quantification of blood ingested by fleas. The capability to detect minute quantities of blood in single fleas, particularly immediately after colonization of the host, will provide a superior tool for studying flea-host interactions, flea-borne disease transmission, and flea control strategies.

## Background

Fleas (*Ctenocephalides *spp.) are the most common ectoparasites of dogs and cats in North America. Although more than 2,200 species and subspecies of fleas are known throughout the world, only *Ctenocephalides felis felis *(cat flea)*, Ctenocephalides canis *(dog flea)*, Pulex simulans*, and *Echidnophaga gallinacea *(poultry sticktight flea) occur in reasonable numbers on pet animals. The most commonly encountered flea species in North America is *C. felis felis *[1-3].

The direct effect of blood consumption in severe flea infestations may be flea allergy dermatitis (FAD), anemia and death [1,2]. An indirect health effect of flea infestations may be the transmission of blood-borne infectious agents such as *Mycoplasma haemofeli, Bartonella *spp., *Rickettsia typhi*, *Rickettsia felis*, and *Yersinia pestis *[4-8]. Yet another outcome is that fleas may serve as intermediate hosts of parasites, such as *C. felis *does for the non-pathogenic subcutaneous filarid nematode of dogs, *Acanthocheilonema *(*Dipetalonema*) *reconditum*, and for several species of cestodes [1,2].

Our current understanding of blood feeding of fleas has remained incomplete because of several experimental limitations: i) studies thus far typically have confined experimental flea inoculations to feeding chambers covering a small, easily accessible host body region rather than allowed for whole body exposure of the host, such that fleas can find their feeding predilection sites; and ii) due to detection limits, many fleas have typically been inoculated into these chambers and blood feeding has been determined for the whole pool. This crowding also may impact flea feeding behavior as much as lack of access to predilection sites does. Collectively, therefore, reliable data on blood feeding of fleas very early after unrestricted access to the host does not exist, and statistical analyses are somewhat unsatisfactory because feeding data exist only for pools, but not for individual fleas.

Accurate determination of the amount of blood consumed by feeding fleas is important for assessing the efficacy of flea control agents and for understanding the role of fleas in the transmission of disease agents, because the efficacy of fleas as vectors depends, in part, on the amount of blood consumption. In particular, quantification of early blood feeding of fleas immediately after contact with a suitable host is essential for accurate evaluation of flea repellents. Methods for detection and quantification of blood include crushing fleas on moist paper, simple weighing of fleas and their feces, radionuclide tags on host erythrocytes (^31^Cr) or ^125^I -albumin [9], and the use of Drabkin's reagent for hemoglobin determination via a cyanmethemoglobin adduct [10].

McCoy et al. (2008) in their study used the cyanmethemoglobin reference procedure for hemoglobin determination and established a linear regression between optical density (OD) and amount of blood. This equation indicates that 1 μL of blood produces double the background OD, indicating that the practical detection limit of the assay is approximately 0.5 μL of blood. They detected an average consumption of 0.17 μL blood by male fleas allowed to feed on cats for 60 minutes, thus requiring pooling of fleas for a stable readout. This is not sensitive enough to quantify the blood feeding patterns of individual fleas, nor the blood feeding patterns of fleas early after host access.

This methodological deficiency prompted us to develop a quantitative PCR for detection of the mammalian homolog of a gene that could be used to quantify blood ingested by fleas with higher sensitivity that is required for analyses of early flea-host interactions, of precise dynamics of flea-borne disease transmission, and of flea control strategies. This PCR targets the HMBS gene, a single-copy gene of the heme synthesis pathway [11]. The HMBS PCR amplifies DNA present in circulating white blood cells acquired by fleas while feeding on dogs, and HMBS gene quantitative detection therefore allows exquisitely sensitive and specific determination of flea blood feeding.

## Methods

### Experimental animals

Eight healthy male and female Beagle dogs of 13-16 months age and weighing 15.4-24.3 lbs were used for establishment and validation of the HMBS qPCR. All dogs were obtained from Ridglan Farms (Mt. Horeb, Wisconsin, USA). Physical examination performed on the dogs upon arrival at the study site confirmed their overall health, including a normal hemogram. All dogs were housed individually in indoor/outdoor kennels that were free of fleas. Water and a commercially available dry ration were provided *ad libitum*. All animal procedures in this study were reviewed and approved by the Auburn University Institutional Animal Care and Use Committee.

### Flea maintenance and challenge

The AuEL laboratory strain of *Ctenocephalides felis *used in this study was insectary-reared and propagated on laboratory cats that are maintained in the laboratory of the investigators. For maintenance of AuEL, larvae are fed *in vitro *on bovine dried blood. Therefore, it was necessary to confirm that blood detected in adult fleas reared from larvae fed on bovine blood did not originate from feeding of larvae *in vitro*. The AuEL strain has been maintained by continuous passage since 1985. Dogs were challenged with 100 adult fleas (1:1 male:female) which were removed by personnel wearing fresh gloves by the use of fine tooth flea combs, and chilled to allow for collection and sexual differentiation. New combs were used for each collection to prevent carry-over of hair, dandruff and skin cells. Fleas were identified to gender based on size and external reproductive organs located on the abdominal body segment of adult fleas.

### Quantitative PCR

#### Design of primers and probes

**Figure 1:**
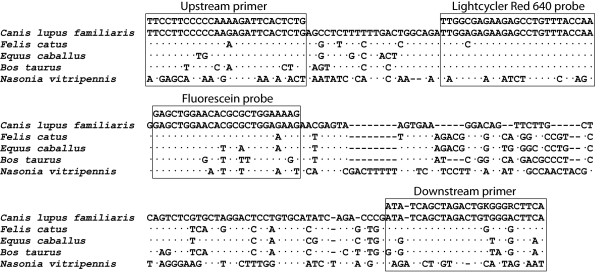
**Alignment of HMBS amplification targets of *Canis lupus familiaris*, *Felis catus*, *Equus caballus*, *Bos taurus*, and jewel wasp *Nasonia vitripennis***. Dots indicate nucleotides identical to the canine HMBS sequence, and dashes represent nucleotide deletions. Primers and probes are shown by boxes. The upstream primer is used as shown while the downstream primer and probes are applied as reverse complement (antisense) oligonucleotides. The probes were designed for discrimination of the homologous sequences of different animal species. K represents G or T, and R stands for A or G.

Nucleotide sequences of the HMBS genes of *Canis lupus familiaris *(NC_006587.2, whole gene region: 17774346-17781513), *Felis catus *(AANG01077049, amplification product region: 15323-15521), *Equus caballus *(AAWR02020903.1, amplification product region: 93984-94182), *Bos taurus *(AAFC03046256.1, region homologous to amplification product: 61960-61765), and the jewel wasp *Nasonia vitripennis *(AAZX01001902.1, region homologous to amplification product: 13757- 13550) were obtained from GenBank. Primers and probes were designed by use of the Vector NTI software (Invitrogen Corporation, Carlsbad, CA, USA) and synthesized by Integrated DNA Technologies (Coralville, IA, USA). The HMBS nucleotide target sequences and positions of primers and probes are shown in Figure 1. The HMBS upstream and downstream primers were placed on introns 4 and 5 (CaFeHMBSUP, 5'-TTCCTTCCCCCAAAAGATTCACTCTG-3'; and CaFeHMBSDN, 5'-TGAAGYCCCMCAGTCTAGCTGATAT-3'), and the HMBS probes were placed on exon 5. The fluorescein probe (CaFeHMBSFLU, 5'-CTTTTCCAGCGCGTGTTCCAGCTC-6-FAM-3') was 3'-labeled with carboxyfluorescein (6-FAM). This probe was used unpurified and acts as a fluorescence resonance energy (FRET) donor probe excited by 488 nm light. The LightCycler Red 640 probe (CaFeHMBSLCR640, 5'-LCRed640-TTGGTAAACAGGCTCTTCTCGCCAA-Phosphate-3') was HPLC-purified and used as FRET acceptor probe, emitting 640 nm fluorescence following excitation by physical proximity to 6-FAM. Primers were designed to amplify DNA of mammalian host species of fleas, including prominently dogs and cats, but not cattle, the blood of which is used for maintenance of flea colonies, or arthropods. The fluorescein probe is also designed for maximum discrimination between these species, while the LightCycler Red probe hybridizes to the known relevant mammalian homologs, but not to arthropod sequences.

#### Extraction of total nucleic acids

Collected fleas were immobilized by brief exposure to -20°C, and immediately put into 2 ml screw-cap microcentrifuge tubes containing 500 μL RNA/DNA stabilization buffer (Roche Molecular Biochemicals, Indianapolis, IN, USA) and 6 ceramic beads of 3 mm diameter (MO BIO Laboratories Inc, Carlsbad, CA, USA). Fleas were homogenized by vigorous shaking (3 × 60 seconds) in a Precellys 24 tissue homogenizer (Bertin Technologies, Saint Quentin en Yvelines, France). Total nucleic acid extraction was performed by glass fiber matrix binding and elution with the High-Pure PCR Template Preparation Kit (Roche Molecular Biochemicals, Indianapolis, IN, USA) as described before [12]. Each sample was eluted in 40 μL elution buffer such that a PCR input of 10 μL was equivalent to 1/4 of the total extracted DNA.

#### Real-time PCR and melting curve analysis

To create the quantitative standards in the HMBS PCR, a 188-bp nucleotide fragment representing the canine HMBS gene sequence was synthesized and inserted in the pIDTSMART cloning vector (Integrated DNA Technologies, Coralville, IA, USA). The plasmid was restricted with *Hind*III (Promega, Madison, WI, USA), and the restriction enzyme was inactivated at 65°C for 20 min. DNA was quantified by PicoGreen^® ^DNA fluorescence assay (Molecular Probes, Eugene, OR, USA) for calculation of the number of target molecules and for preparation of quantitative standards.

**Figure 2:**
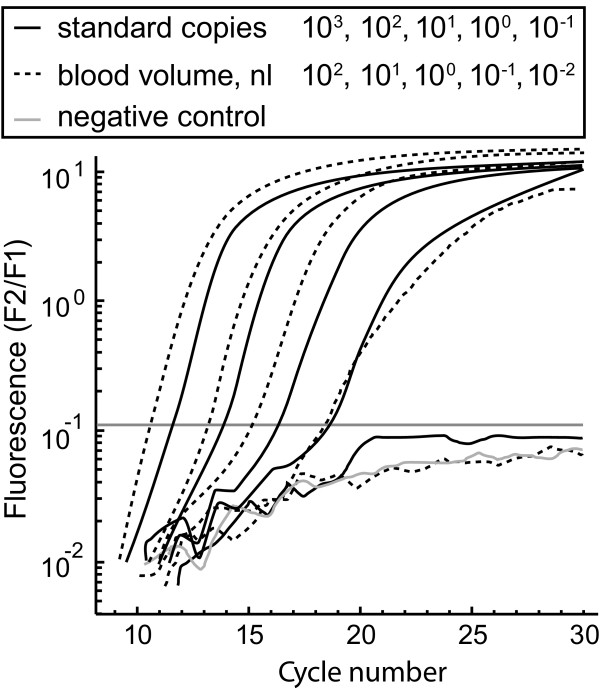
**PCR amplification curves of HMBS standards and canine blood specimens**. HMBS standards and DNA of a canine whole blood specimen were logarithmically diluted (10^3 ^to 10^-1 ^HMBS template copies or PCR input equivalents of 10^2 ^to 10^-2 ^nL canine blood) were amplified in a background of 200 ng pUC19 plasmid DNA. Fluorescence channel F2 normalized by F1 (F2/F1) shows the robust amplification curves of HMBS standards and canine whole blood specimens. The 10^3 ^copies or 10^2 ^nL blood amplification curves cross the fluorescence threshold at approximately cycle 10, while the 10^0^ copy or 10^-1 ^nL curves (single copy or 0.1 nL blood) cross at approximately cycle 19. In contrast, the 10^-1 ^copy or 10^-2 ^nL curves (0.1 theoretical copy or 0.01 nL blood) do not cross the threshold and are indistinguishable from the negative control. On average for all amplifications, the nL amount of blood was equivalent to 23 × the amount of HMBS copies detected, defining the theoretical detection limit (without consideration of Poisson sampling error) as 0.043 nL blood per 1 HMBS copy.

The HMBS copy number was determined in FRET-PCR performed on a LightCycler^® ^1.5 real-time PCR platform with Software version 3.53 (Roche Molecular Biochemicals, Indianapolis, IN, USA) with PCR conditions as previously described [12]. The sensitivity of the PCRs was confirmed by amplification of logarithmic dilutions of HMBS standard and DNA extracted from canine blood (Figure 2). For specificity assurance, the HMBS PCR was performed on nucleic acids extracted from adult fleas reared from larvae fed on bovine blood, and from bovine, canine, equine, feline, and porcine blood followed by melting curve analysis. PCR products were verified by automated DNA sequencing of both strands at the Genomic Sequencing Laboratory (Auburn University, Auburn, AL).

### Statistical analysis

**Figure 3:**
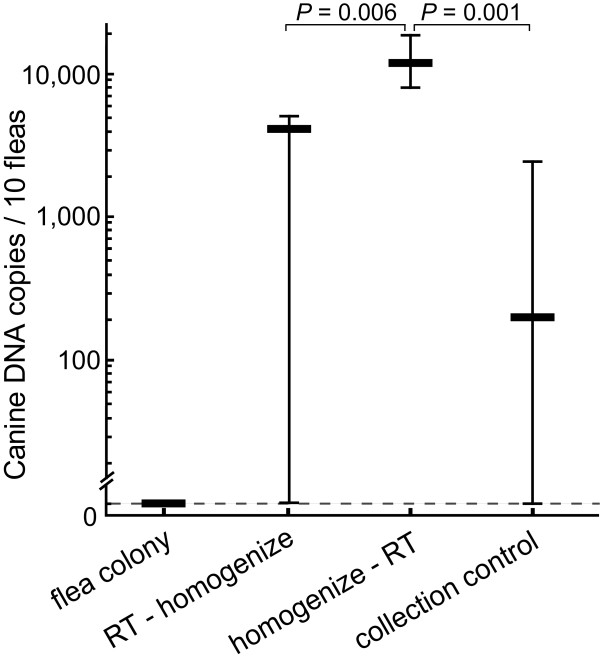
**Quantification accuracy of canine blood ingestion by fleas**. HMBS copies were quantified for individual fleas fed only bovine blood that were collected directly from the flea colony followed immediately by homogenization and nucleic acid extraction (flea colony; n = 10 fleas). Another group contained fleas that were immediately removed from the dog and DNA was immediately extracted (collection control; n = 16). For evaluation of HMBS DNA recovery, fleas collected after 4 hours on the dog were either stored for 7 days at room temperature in RNA/DNA Stabilization buffer followed by homogenization (RT - homogenize; n = 10) or were homogenized immediately after collection followed by 7-day RT storage (homogenize - RT; n = 10). HMBS amplification was not observed in fleas from the colony, while fleas in the collection control group did contain a small but significant amount of HMBS targets indicating a background level of contamination with canine DNA by contact of the fleas with the dog. Fleas of the homogenize-RT group carried significantly higher HMBS copy numbers than RT-homogenize fleas (*P *= 0.006) indicating incomplete preservation of DNA in non-homogenized fleas. Fleas of the homogenize-RT group (the final collection approach) contained highly significantly more HMBS copies than those of the collection control group (*P *= 0.001), indicating a significant amount of blood feeding. Thick bars indicate the mean HMBS, and error bars indicate minimum/maximum copies per 10 fleas.

**Figure 4:**
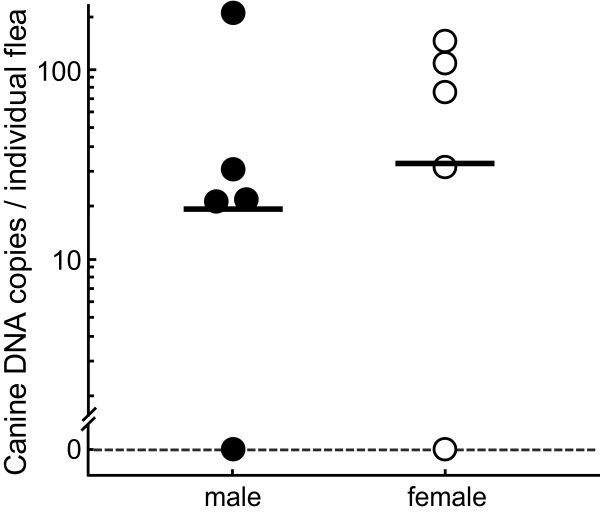
**Ingestion of canine blood by individual male and female fleas**. Mixed populations of male and female fleas were removed from a dog after 15 minutes. DNA was extracted from individual fleas, HMBS copies per flea were determined, and an HMBS background of 31.5 copies per flea was subtracted. Solid circles indicate male fleas, and open circles female fleas. Bars indicate the logarithmic mean of HMBS copies per group (n = 5). The number of HMBS copies in male (19.8) and female (31.2) fleas do not differ significantly (*P *= 0.53, Mann-Whitney U-test).

**Figure 5:**
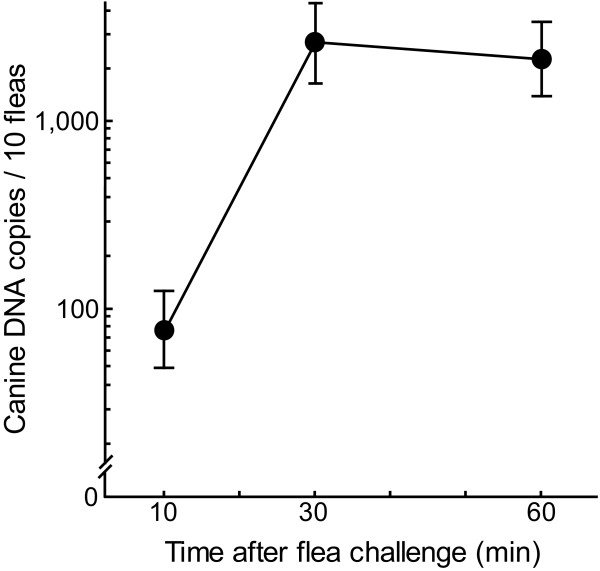
**Time-course of canine blood ingestion by fleas**. Fleas were put on eight dogs, and 40 fleas were removed from each dog at the indicated time points after challenge. DNA was extracted from pools of 10 fleas (4 pools per dog and time point), HMBS targets were quantified by PCR (means ± 95% CI), and an HMBS background of 315 copies per 10 fleas was subtracted. HMBS copies per 10 fleas collected after 10 min on the dogs were significantly lower than those for 30 and 60 min (74 vs. 2703 and 2225 copies; *P *= 0.0001, Tukey HSD test).

All statistical analyses were performed with the Statistica 7.1 software package (StatSoft, Inc., Tulsa, OK, USA). The background HMBS copies (31.5 copies/flea, or 315 copies/pool of 10 fleas) were subtracted from the original HMBS PCR data, and the data were logarithmically transformed. The Mann-Whitney U test was used to analyze HMBS copies in determination of quantification accuracy of canine blood ingestion by fleas (Figure 3) and of the ingestion of canine blood by individual male and female fleas (Figure 4). PCR data of the time-course of canine blood ingestion were analyzed by one-way ANOVA and Tukey HSD tests (Figure 5). Differences at *P *≤ 0.05 were considered significant.

## Results

### Establishment and validation of the HMBS PCR

#### Specificity

The HMBS PCR was designed to amplify canine and feline HMBS targets efficiently and robustly, but not other mammalian or arthropod target HMBS genes. It amplifies the equine target much less efficiently (6-bp mismatches with primers) and the fluorescent signal is delayed by approximately 15 thermal cycles. The bovine (9-bp mismatches with the primers) or porcine as well as the flea homologues were completely refractory to amplification. Following the completion of the PCR, melting curve analysis allowed a second level of differentiation. The feline HMBS gene showed a higher probe melting point (no mismatch, *T_m _*= 63.8°C) than the canine gene (1-bp mismatch on each probe, *T_m _*= 58°C) or the equine gene (2-bp mismatches on the fluorescein probe, *T_m _*= 61.5°C). These *T_m _*differences allowed unambiguous differentiation of the host species of amplified HMBS targets.

#### Sensitivity

PCR amplification of logarithmic dilutions of HMBS standards and canine whole blood confirmed the high sensitivity and robustness of this PCR. Logarithmic dilutions of the HMBS standard DNA (10^3^, 10^2^, 10^1^, 10^0^, and 10^-1 ^template copies) and of total nucleic acids extracted from canine whole blood containing 9,800 white blood cells per microliter (10^2^, 10^1^, 10^0^, 10^-1^, and 10^-2 ^nL canine blood) were amplified in a background of 200 ng pUC plasmid DNA. Fluorescence channel F2 normalized by F1 (F2/F1) shows the robust amplification curves of HMBS standards and canine blood (Figure 2). The amplification curve of the HMBS standard with 10^3 ^target copies crosses the fluorescence threshold at approximately cycle 11.5 while the amplification curve of 10^2 ^nL blood crosses at approximately cycle 10.1, equivalent to approximately 2,300 copies of the HMBS gene. The 10^0 ^standard copy or 10^-1 ^nL blood sample reactions (one HMBS copy or 0.1 nL blood) showed highly robust PCR amplification curves, while the 10-fold lower dilutions (0.1 copy or 0.01 nL) do not cross the fluorescence threshold (Figure 2). These data established that the PCR is equally effective at high copy number or single copy input, and verified the robust ability of the HMBS PCR to detect single target copies, equivalent to approximately 0.043 nL of canine whole blood.

### Flea collection, nucleic acid preservation and extraction

To fully verify the rationale of this study and further ensure the specificity of the HMBS PCR, we first evaluated amplification of the HMBS target in a group of 10 bovine blood-fed fleas that were collected directly from the maintenance insectory. As expected, HMBS amplification was not observed in these fleas (Figure 3).

Since the HMBS PCR detects any canine genomic DNA, we next evaluated the amount of canine DNA acquired by mere contact of fleas with dogs without any possibility for blood feeding. Fleas were put on dogs, allowed to distribute in the hair for approximately 10 seconds, and then immediately combed off with new combs. HMBS amplification was observed in 12 of 16 fleas of this collection control group, averaging 21 HMBS copies per flea, with a standard deviation of 10.5 copies (Figure 3). This indicated a background level of contamination with canine DNA by contact of the fleas with the dog, and 315 HMBS copies per 10 fleas (logarithmic mean + SD) were subtracted from all sample data in this study.

Efficient extraction and appropriate preservation of nucleic acid is essential for PCR amplification, and this might be particularly important for fleas. The rigid exoskeleton structure of fleas resists grinding as well as penetration by guanidinium, nucleic acid preservation buffer, and therefore potentially compromises preservation and extraction of nucleic acids. To analyze DNA preservation and recovery, fleas were put on dogs for 4 hours and subsequently stored in RNA/DNA stabilization buffer at room temperature for 1 week in 20 groups of 10 fleas. Fleas in 10 of the groups were immediately homogenized by shaking in the Precellys 24 homogenizer (homogenize-RT, Figure 3), while 10 groups were homogenized after storage (RT-homogenize, Figure 3). Fleas from the homogenized-RT group showed significantly higher HMBS copy numbers than RT-homogenized fleas (Figure 3). This indicates incomplete preservation of DNA in the fleas that were not homogenized immediately after collection, probably due to incomplete penetration of the fleas by the stabilization buffer. Based on this result, immediate homogenization of the fleas was adopted as a standard protocol in this study.

### Blood feeding behavior of male and female fleas

To evaluate potential sex differences in the feeding behavior of fleas, mixed populations of male and female fleas were removed after 15 minutes on a dog. Fleas were immobilized by brief placement at -20°C and gender was determined microscopically in the cold room with fleas placed on separate DNA-free microscope slides, and five individual male and female fleas each were placed in separate tubes containing RNA/DNA stabilization buffer and immediately homogenized for DNA extraction and HMBS PCR (Figure 4). HMBS copies did not differ significantly between male and female fleas (19.8 vs. 31.2; Mann-Whitney U-test). The data furthermore show the high variance of HMBS copies in both male (0 to 216.6) and female fleas (0 to 134.3). This indicates that some fleas feed quickly on dogs, while others do not, and that this behavior does not differ between male and female fleas.

### Temporal dynamics of flea feeding

To conduct an initial analysis of the time course of early blood feeding, fleas from the insectory were put on eight dogs, and 40 fleas were removed in separate experiments 10, 30 or 60 minutes after challenge (4 pools of 10 fleas/dog/time point) (Figure 5). Ten minutes after exposure to the dogs, 10 fleas contained 74 copies of HMBS. This number is significantly lower than the HMBS copies at 30 min (2703) or 60 min (2225) (*P *= 0.0001, Tukey HSD test). These results suggest that fleas do not start blood feeding immediately upon contact with a dog. The amount of the blood consumed per flea at 60 min is approximately 100 nL, when a ratio of 23 for HMBS copies to nL blood volume is assumed (Figure 2).

## Discussion

In this study, we developed a novel approach to quantify flea blood feeding on mammals by highly sensitive and accurate determination of host DNA within the fleas. A segment of the HMBS gene, an evolutionary divergent gene, was amplified by quantitative PCR which also determined the host species by the FRET probe melting temperature. Limiting dilution of DNA templates as well as canine blood confirmed that this PCR has a detection limit of a single mammalian genome present in circulating white blood cells, equivalent to approximately 0.043 nL blood or less in animals with physiological white blood cell counts (~10,000 white blood cells/μl blood). This is an approximately 10,000-fold higher sensitivity than the standard cyanmethohemoglobin method employed by McCoy et al. [10]. Differential sample collection and storage testing, negative testing of fleas from the insectory fed only bovine blood, and detection of small quantities of canine DNA on fleas that had short contact with dogs without blood feeding, established a viable methodology and determined the practical DNA background of the method.

The capability to detect small quantities of blood in single fleas, particularly within minutes of colonization of the host, will provide a necessary tool for studying flea-host interactions. In our validation analyses we showed that there was no statistical difference between male and female fleas in the quantity of approximately 1.1 nL of blood and/or canine epithelial cells (25.5 HMBS copies × 0.043) consumed within 15 minutes after host contact, but a high variance in the amount detected per flea. Also, in the preliminary analysis of the time course of blood feeding, fleas contained on average approximately 0.3 nL of blood at 10 minutes after exposure to the dogs, but highly significantly more at 30 and 60 min. These results suggest that fleas quickly "sample" the dog, but do not start continuous saturation blood feeding immediately upon contact with a dog. Cadiergues et al. [13,14] studied initiation and duration of flea feeding on dogs and cats, and demonstrated that 20% of the fleas on dogs initiated feeding within 5 minutes, ~50% between 15 and 30 minutes, and 71% by 1 hour, without difference between male and female fleas. These data are consistent with our limited sample. However, Cadiergues et al. [13,14] used only qualitative detection of the blood meal by microscopic examination of the fleas, and therefore could not differentiate quick aberrant sampling behavior from saturation blood feeding. This differentiation may prove important in the evaluation of flea control strategies and their effect on transmission of vector borne diseases [7], but larger studies need to confirm these initial observations obtained with a relatively small numbers of fleas. Another interesting observation is the fact that the canine blood content of fleas of ~118 nL at 30 min after contact dropped to ~100 nL at 60 min. This may be the consequence of establishment of a steady-state equilibrium between blood uptake and removal in the fleas by digestion and/or fecal excretion of blood.

An important difference between this study and previous studies is that fleas were placed on dogs by application to the dorsal midline haircoat. Previous studies utilized entrapment devices into which fleas were placed [10]. Although this strategy allows for accurate recovery and enumeration of fleas and quantification of flea feces, it likely imposes behavioral changes in flea feeding due to the high population densities of fleas in the enclosures. We propose that the high sensitivity of the HMBS PCR will also be helpful in studying flea early blood feeding in close simulation to the natural behavior of fleas without the use of entrapment devices required for flea pooling.

In this study, we established the HMBS PCR method and conducted small, preliminary experiments that validated the assay. We used blood from a single dog with a standard number of ~10,000 white blood cells per microliter of blood to establish a correlation between HMBS gene copy numbers and blood volume (Figure 2). In subsequent experiments, we analyzed repetitive treatment groups from the same dogs, and could therefore validly use HMBS copy data for statistical analysis of treatment effect (Figures 3, 5). However, in future studies accurate comparisons of fleas collected from different dogs in each treatment group, or conversion of HMBS copy numbers into blood volume, will require consideration of leukograms and correction for different leukocyte numbers of the dogs.

Since the HMBS PCR amplifies not only DNA from leukocytes, but any mammalian DNA, it is important to determine the amount of background signal that is derived exclusively from contact of the fleas with the host. We assume that a host DNA contamination equilibrium becomes established at which new external contamination of fleas is balanced by shedding of prior contamination. In this study, the background per flea was 31.5 HMBS copies, but this may be different for other dogs, housing and feeding conditions, and other personnel. DNA background determination should therefore be performed prior to actual experiments for every dog in a study with a pooled sample of at least 20 fleas collected by the experimental personnel. It will also be important to minimize carry-over contamination with hairs and dandruff of animals while collecting fleas by single use of combs and gloves as in this study.

## Conclusions

The HMBS PCR method developed in the current study offers the advantages of both exquisite sensitivity and specificity that make it superior to other approaches for quantification of blood ingested by fleas [15]. This sensitivity and specificity will be required to further expand our knowledge about flea-host interactions, transmission of flea-borne diseases, and control of flea infestations. Studies are underway to determine the utility of this assay in demonstrating that flea control products, particularly those that act quickly on adult fleas, can greatly reduce feeding and thus reduce the severity of flea-associated diseases.

## Competing interests

This research was funded in part by grants from CEVA Animal Health to Drs. Kaltenboeck and Blagburn. The authors declare no conflict of interest.

## Authors' contributions

CW, BLB, and BK designed the whole experiment. JM and JB performed flea maintenance and challenge. CW, EJ, and DG extracted DNA. CW, BK, and DG designed the PCR, performed calibration experiments, and analyzed the data. BK, CW, and BLB wrote this manuscript. All authors read and approved the final version of the manuscript
